# Modified Y-splitting Procedure for the Treatment of Duane Retraction Syndrome

**DOI:** 10.4274/tjo.70188

**Published:** 2015-08-05

**Authors:** Ayşe Gül Altıntaş, Hasan Basri Arifoğlu, Şükrü Gültekin Köklü

**Affiliations:** 1 Ulucanlar Eye Research and Education Hospital, Ankara, Turkey; 2 Kayseri Research and Education Hospital, Clinic of Ophthalmology, Kayseri, Turkey

**Keywords:** Duane retraction syndrome, downshoot, upshoot, globe retraction

## Abstract

**Objectives:**

To present the outcomes of modified lateral rectus Y-splitting combined with either unilateral or bilateral horizontal rectus recession in Duane Retraction Syndrome (DRS) with significant upshoot or downshoot.

**Ma­te­ri­als and Met­hods:**

A total of 12 patients including 10 patients with Type I DRS and 2 with Type III DRS underwent modified Y-splitting surgery. Amount of additional recessions varied with the degree of preoperative deviation by intraoperative adjustable suture technique. Preoperatively 3 patients had esotropia (ET), 6 had exotropia (XT), and 3 patients had orthotropia. The mean preoperative deviation was 19.3 prism diopters (PD) (range, 18-20 PD) in ET patients and 19.2 PD (range, 16-20 PD) in XT patients.

**Re­sults:**

Postoperatively, all patients had significant correction in horizontal deviation and aligned within 4 PD of orthotropia, and no patients exhibited abnormal head posture. Co-contraction and globe retraction were markedly reduced and abnormal ocular vertical movement disappeared or significantly decreased in all cases. No patients experienced recurrence of ocular motility disorders in the mean 26-month (range, 13-66 months) follow-up period.

**Conclusion:**

Modified Y-splitting surgery combined with co-contracting horizontal muscle recession technique seems to be a safe and effective treatment in DRS.

## INTRODUCTION

Duane retraction syndrome (DRS) is an unusual congenital form of strabismus due to aberrant ocular innervation and is characterized by horizontal eye movement limitation, globe retraction with palpebral fissure narrowing on attempted adduction, and a sharply oblique movement of the affected eye either up and in or down and in on adduction. This condition results from paradoxical anomalous lateral rectus (LR) innervation from the branches of the third nerve due to misdirection of axons destined for the medial rectus (MR). Therefore, the LR muscle is innervated by branches from the oculomotor nerve instead of the abducens. That misdirection causes paradoxical contraction of LR during adduction, meaning Hering’s Law does not apply to DRS.^1,2,3,4,5,6^

**Figure 1:**
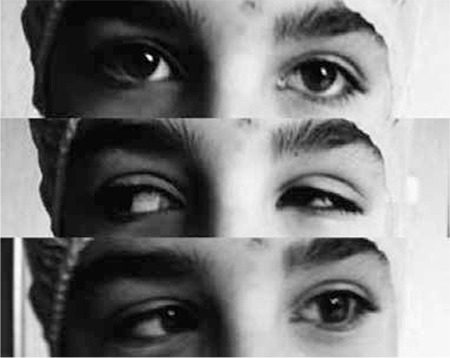
Preoperative ocular movements: primary position; upshoot in adduction and severe globe retraction; abduction deficiency

Indications for surgery in DRS are significant deviation in primary position, significant horizontal deviation in primary position, noticeable abnormal head position (AHP), marked globe retraction and cosmetically unacceptable upshoot or downshoot on attempted adduction (Figure 1).^3,7,8^

Several surgical options have been proposed including recession of the ipsilateral MR or simultaneous MR and LR recession, vertical rectus muscle transposition, posterior fixation sutures, weakening of the LR with either attachment to the lateral canthal tendon or orbital periosteum, and recession and Y-splitting of the LR muscle.^1,3,7,8^

Upshoot and downshoot as a vertical excursion of the globe is believed to be caused by tautness of the lateral rectus muscle due to the absence of normal neural inhibition, or increased paradoxical innervation that causes co-contraction of both horizontal rectus muscles in adduction.^2,3,4,5,6,7^

The aim of this study is to present modified Y-splitting and recession of the lateral rectus with or without simultaneous ipsilateral MR recession to treat severe up and downshoot associated with globe retraction.

## MATERIALS AND METHODS

The study included 12 patients with DRS who had a main symptom of significant functionally and cosmetically unacceptable up or downshoot and globe retraction, and were treated surgically. All patients signed an informed consent approved by the institutional review board.

In addition to full ophthalmological examination including assessment of visual acuity and binocular single vision, motility examination was performed with prism and alternate cover tests. Both horizontal and vertical version and duction were documented on a six-point scale in which +4 is marked over-correction, 0 equals full movement, -4 is unable to move past the midline and -5 is severe motility limitation in which the affected eye was unable to move to the midline.^5^

**Figure 2:**
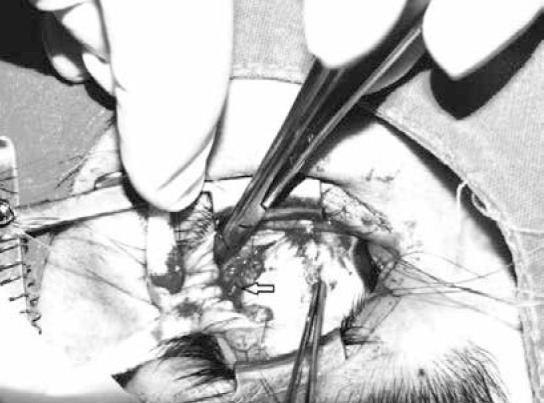
Y-splitting on lateral rectus muscle. Arrow indicates separation suture

All patients underwent LR recession combined with modified Y-splitting performed by the same surgeon, similar to standard Y-splitting. The LR was separated into two halves as far back as possible from the insertion and two double-armed polyglactin sutures were placed into both the upper and lower halves of the insertion and secured with locking bites at each end of the separated muscle (Figure 2). The LR was disinserted and recessed; to create the Y configuration, the recessed superior half of the muscle was inserted into the sclera in line with the superior border of the original insertion and the recessed inferior half was sutured to the sclera in line with the inferior border. In modified Y-splitting, in addition to the standard procedure described above, non-absorbable separation sutures were placed around the split parts of each half without scleral fixation in order to prevent reproliferation and refusion of the middle split.^3^

Patients with horizontal deviation associated with AHP and severe globe retraction underwent additional MR recession. The MR and LR were recessed approximately the same amount in patients with preoperative orthophoria in primary position. The MR was recessed relatively more than the LR in cases of esotropia (ET) and the LR was recessed relatively more than the MR in cases of exotropia (XT) in primary position. Patients older than 15 years underwent surgery with intraoperative adjustable suture technique.^9^ In addition to degree of preoperative deviation, intraoperative forced duction test and ocular motilities were evaluated to decide the amount of recession.

**Figure 3:**
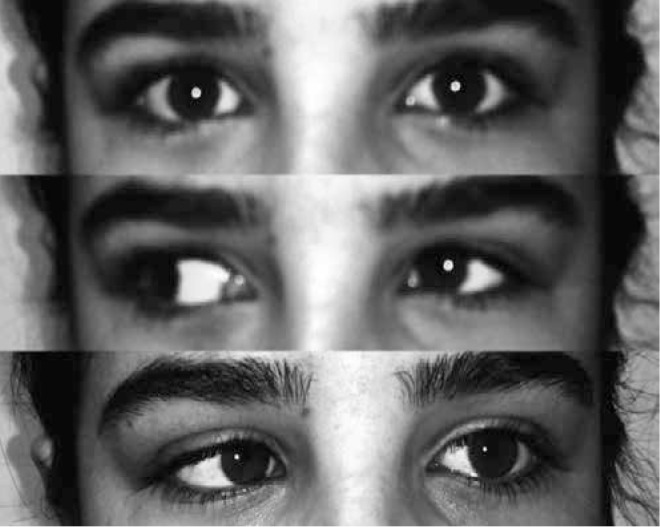
Ocular movements 18 months after surgery

Success was defined as correction of horizontal deviation, elimination of the excursion of the adducted eye in elevation or depression, and diminished globe retraction (Figure 3).

## RESULTS

Among the 12 DRS patients, 8 were female and 4 were male. The mean age was 14.6 years (range, 6-50 years) at surgery. The average follow-up period was 26 months (range, 13-66 months). Ten patients had type I DRS, 2 had type III DRS and no patients had type II DRS. A total of 3 patients had ET, 6 had XT, and 3 patients had orthotropia. The mean preoperative deviation was 19.3 PD (range, 18-20 PD) in ET cases and 19.2 PD (range, 16-20 PD) in XT cases. All of the 12 patients had significant up or downshoot and cosmetically unacceptable globe retraction on adduction.

All patients underwent modified Y-splitting with separation suture in both upper and lower halves combined with LR recession of 4 to 7 mm from insertion and MR recession of 4 to 6 mm based on preoperative deviation, degree of globe retraction and upshoot or downshoot.

None of the cases exhibited postoperative improvements in abduction; postoperative adduction was also essentially unchanged. Up and downshoot disappeared completely in 8 cases and significantly decreased in 4 cases. Globe retraction was eliminated in 9 cases and diminished in 3 cases. AHP was corrected in all patients and orthotropia was achieved in 10 cases; 4 PD XT persisted in 2 cases. During the follow-up period, none of the patients had persistent or recurrent AHP, up/downshoot, or globe retraction similar to the preoperative period.

## DISCUSSION

A total of 12 cases were successfully treated by modified Y-splitting and horizontal muscle recession surgery by eliminating up and downshoot and globe retraction and correcting horizontal deviation. DRS is the most common type of congenital ocular innervation abnormality, accounting for 1-4% of all strabismus cases; however, most patients compensate well for the disorder, and therefore do not require surgical management. Extraocular muscle surgery does not eliminate the abnormality of muscle innervation and no surgical technique can successfully restore normal ocular movement, as we observed in our cases.^2,3,4,7,8^

Our primary aims of surgery are to restore ocular alignment in primary position, relieve AHP, eliminate narrowing of the palpebral fissure with retraction of the globe and treat up or downshoot, which was the most prominent feature of our cases.

The up or downshoot combined with globe retraction is due to the secondary tautness of LR caused by co-contraction of both MR and LR on attempted adduction. We preferred LR recession of 4 to 7 mm with modified Y-splitting for treatment of anomalous vertical movement in adduction and combined it with MR recession of 4 to 6 mm to eliminate globe retraction. Recession of both horizontal recti is done to decrease the tension on the globe by weakening co-contracting muscles. The surgical procedure was modified slightly according to forced duction test and intraoperative ocular alignment in cases that underwent intraoperative adjustable suture technique. Nearly all of our patients achieved orthotropia and none had persistent AHP.

Sachdeva et al.^10^ reported an average 5.5 to 5.7 mm MR recession with or without LR recession in 14 patients with ET DRS and achieved 85.7% success in correcting ET; they also observed satisfactory improvement in associated globe retraction and AHP. Consistent with the results of that study, our observations also support that weakening co-contracting muscles is effective in eliminating globe retraction.

Naton et al.^11^ reported that an average 6.3 mm unilateral single horizontal rectus muscle recession decreased strabismus to less than 10 PD in 85% and eliminated or improved head turn to less than 10 degrees in 93% of cases. Their average recession was greater than in our and the Sachdeva et al.^10^ series. Naton et al.^11^ also reported a high success rate with large single muscle surgery. Okumuş et al.^12^ observed significant correction of AHP after single muscle recession in type I DRS.

These results show that different types of horizontal rectus recession surgeries are effective procedures for the treatment of clinical findings due to co-contraction.

In DRS with severe up and downshoot, a Y-splitting of the ipsilateral LR is generally recommended. Velez et al.^13^ performed isolated Y-splitting combined with a mean LR recession of 8.7 mm in 10 XT DRS patients with limitation of adduction and up or downshoot. They observed a significant decrease in downshoot and a trend towards improvement of upshoot, but 3 out of 10 patients required additional surgeries. Rao et al.^14^ performed recession and Y-splitting of the lateral rectus in 10 patients with DRS; 6 patients out of 10 also had MR recession simultaneously to correct globe retraction and ocular deviation. All patients in their series showed a marked decrease in up or downshoot as well as improvement in globe retraction.

We performed modified Y-splitting with separation suture in both upper and lower halves combined with recession of one or both horizontal recti. Our success rate was slightly higher than other authors’. We did not observe any complications such as restricted adduction or anterior segment ischemia, which have been reported by other authors.^15,16,17^ The key to our success was forced duction testing during the operation to confirm relief of the mechanical factor and estimate both postoperative ocular motility and deviation with the help of intraoperative adjustable sutures.

She et al.^18^ performed both superior and LR recession in 5 cases with type III DRS with globe retraction, 3 of whom had vertical deviation in primary position. They observed reduction of vertical deviation in all cases but development of subsequent ET in one patient. Our two patients with type III DRS with significant globe retraction underwent modified Y-splitting and recession of both horizontal recti; upshoot disappeared postoperatively in both cases and neither developed subsequent deviation.

Only one patient who underwent classical Y-splitting redeveloped upshoot and globe retraction due to fibrous proliferation in the middle of the LR split three months postoperatively. Therefore, to prevent refusion in this patient, a modified Y-splitting procedure was developed in which non-absorbable separation sutures were placed around the two halves without scleral fixation.^3^ Another 11 patients underwent modified Y-splitting in their initial operation. We did not observe any recurrence during the follow-up period of up to 66 months, which to our knowledge is the longest regular follow-up period of the Y-splitting procedure.

To the best of our knowledge, our case series is the largest series of DRS surgeries performed by the same surgeon, and it also demonstrates the long-term effectiveness of the modified Y-splitting procedure in preventing recurrence.

According to our results, modified Y-splitting combined with recession of one or both horizontal recti is an effective procedure in the treatment of significant up and downshoot associated with globe retraction in DRS, with or without horizontal deviation.
